# Transient experimental demonstration of an elliptical thermal camouflage device

**DOI:** 10.1038/s41598-017-17016-7

**Published:** 2017-11-30

**Authors:** Xiao He, Tianzhi Yang, Xingwei Zhang, Linzhi Wu, Xiao Qiao He

**Affiliations:** 1 Key Laboratory of Advanced Ship Materials and Mechanics, College of Aerospace and Civil Engineering, Harbin Engineering University Harbin, Harbin, 150001 PR China; 2 Department of Civil and Architectural Engineering, City University of Hong Kong, Tat Chee Avenue, Hong Kong; 3 Faculty of Aerospace Engineering, Shenyang Aerospace University, Shenyang, 110136 PR China; 4 Center for Composite Materials, Harbin Institute of Technology, Harbin, 150001 PR China

## Abstract

The camouflage phenomenon (invisibility or illusion) of thermodynamics has attracted great attentions and many experimental demonstrations have been achieved by virtue of simplified approaches or the scattering cancellation. However, all of the experiments conducted are limited in the invisibility of spheres or two-dimensional (2D) cylinders. An ellipsoid camouflage device with a homogenous and isotropic shell is firstly reported based on the idea of the neutral inclusion and a 2D elliptical thermal camouflage device is realized by a thin-layer cloak of homogeneous isotropic material firstly. The robustness of this scheme is validated in both 2D and 3D configurations. The current work may provide a new avenue to the control of the thermal signatures and we believe this work will broaden the current research and pave a new path to the control of the path of the heat transfer.

## Introduction

Due to the fantastic performance and significant impact on science and engineering, the camouflage phenomenon (invisibility or illusion) has attracted great attention from the earliest times to the present day. Benefiting from the transformation optics (TO) proposed by Pendry^1^, many kinds of cloaking devices have been achieved in theory and realized in experiment, not only for optics but also for electromagnetic waves, for acoustic and elastic waves, and for heat flux (see, e.g., refs^2–22^ for a sampling). Recently, a multi-physics cloaking approach that can simultaneously manipulate the electric current and the thermal field in the steady state has introduced new excitement into this field^23–25^.

Although the TO method has rapidly established itself as a very powerful, systematic and versatile approach to manipulating different physical quantities and has been successfully generalized into thermodynamics^10–12,15,18,22^, the proposed thermal camouflage devices^22^ that are based on TO demand complex, anisotropic distributions of thermal conductivity, which makes experimental realizations challenging and complicated. To simplify the experiments required, a new method based on searching for the analytical solution of the steady-state heat conduction equation was proposed; then, a bilayer and an ultrathin spherical thermal cloak were demonstrated experimentally based on new methods in refs^19,21^, respectively. Recently, these theoretical methods were summarized into the concept of scattering cancellation^26^, which was previously proposed in the field of electromagnetic waves. To date, it looks like that the preparation of a thermal cloak can be simplified and that the cloak can be made flexible enough due to its homogenous and isotropic property; however, the experiments already carried out are limited to the invisibility of spheres or two-dimensional (2D) cylinders, which cannot satisfy applications in physical space. Consequently, even a small modification of the cloak’s geometry, for example, just a change from sphere to ellipsoid, will make substantial development and will mark a new beginning of the age of versatile research in thermal camouflage. Hereafter, we propose a general method to design an ellipsoidal thermal camouflage device with a homogeneous and isotropic coating based on the intriguing idea of neutral inclusion. In addition, we report the first realization of a 2D elliptical thermal camouflage device based on a thin-layer cloak of homogeneous isotropic material. The robustness of this scheme is validated in both 2D and 3D configurations.

## Results

**Figure 1:**
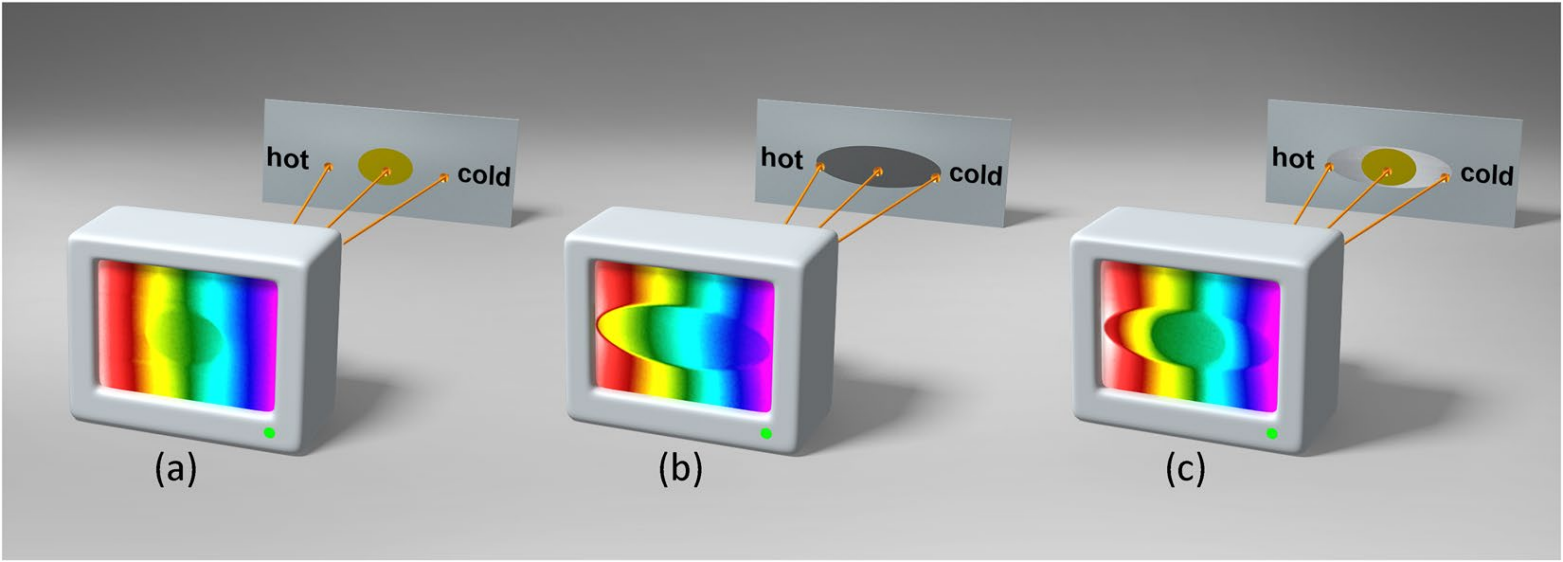
Illustration of an Elliptical Thermal Camouflage Device. The Thermal Signal Shown in (**a**) of an alloy plate including elliptical Cu is completely different from that shown in (**b**) of an alloy plate including elliptical Pb. However, this situation is entirely changed after cloaking the Cu with an elliptical steel shell shown in (**c**), where the thermal signal is exactly the same as the one shown in (**b**) outside the object.

In this paper, we report a meaningful functional thermal camouflage device (see Fig. 1) that transforms the thermal scattering signature of an object into other expected objects with different material parameters. In other words, the functional device can make the thermal scattering signature of the “cloaked” object equivalent to that of another interfering medium. Different from the previous transformation optics-based proposals that require complicated metamaterial designs, thereby leading to many practical limitations, our thermal camouflage device is designed on the basis of one isotropic homogenous layer of cloak derived from the idea of neutral inclusion and only employs naturally occurring materials. In addition, corresponding simulations and experiments that use several common materials are carefully conducted, demonstrating the excellent thermodynamic performance of the proposed approach.

Consider a coated ellipsoid that has a core of phase I with a homogeneous isotropic thermal conductivity *κ*_I_, surrounded by a coating of phase II with a homogeneous isotropic thermal conductivity *κ*_II_. The semi-axis lengths of the core and exterior surfaces of the coated ellipsoid are *l*_*cj*_ and *l*_*ej*_ (*j* = 1, 2, 3), respectively. In this paper, the coated ellipsoid is located in a matrix material with a homogeneous isotropic thermal conductivity*κ*_0_.

In ref.^27^, the effective conductivity tensor $${{\boldsymbol{\kappa }}}_{\ast }=diag[\begin{array}{ccc}{\kappa }_{1}^{\ast } & {\kappa }_{2}^{\ast } & {\kappa }_{3}^{\ast }\end{array}]$$ of the coated ellipsoid was derived as follows:

1 $${f}_{{\rm{I}}}{\kappa }_{{\rm{II}}}{({{\boldsymbol{\kappa }}}_{{\boldsymbol{\ast }}}-{\kappa }_{{\rm{II}}}{\bf{I}})}^{-1}={{\kappa }}_{{\rm{II}}}{({{\kappa }}_{{\rm{I}}}-{\kappa }_{{\rm{II}}})}^{-1}{\bf{I}}+({{\bf{D}}}_{c}-{f}_{{\rm{I}}}{{\bf{D}}}_{e})$$

where ${f}_{{\rm{I}}}=\frac{{l}_{c1}{l}_{c2}{l}_{c3}}{{l}_{e1}{l}_{e2}{l}_{e3}}$ is the volume fraction of the core, and where $${{\bf{D}}}_{c}=diag[\begin{array}{ccc}{d}_{c1} & {d}_{c2} & {d}_{c3}\end{array}]$$ and ${{\bf{D}}}_{e}=diag$ $$[\begin{array}{ccc}{d}_{e1} & {d}_{e2} & {d}_{e3}\end{array}]$$ are the depolarization tensors of the core and the exterior surfaces, respectively, of the coated ellipsoids. The depolarization factors ${d}_{j}({l}_{1},{l}_{2},{l}_{3})$ (*j* = 1,2,3) always sum to unity and can be derived by Eq. (7.53) given in ref.^27^.

For a sphere (*l*_1_ = *l*_2_ = *l*_3_), a cylinder (*l*_1_ = *l*_2_,*l*_3_ → ∞), a spheroid (*l*_1_ = *l*_3_) and an elliptical cylinder (*l*_3_ → ∞), the expressions of the depolarization factors reduce to

2a $${d}_{1}={d}_{2}={d}_{3}=1/3,{\rm{for}}\,{\rm{sphere}}$$

2b $${d}_{1}={d}_{2}=1/2,{d}_{3}=0,\,{\rm{for}}\,{\rm{cylinder}}$$

2c $$\begin{array}{c}{d}_{1}=1-2{d}_{2}=\frac{1-{\varepsilon }^{2}}{{\varepsilon }^{2}}\{\frac{1}{2\varepsilon }\,\mathrm{ln}(\frac{1+\varepsilon }{1-\varepsilon })-1\},\,\varepsilon =\sqrt{1-{({l}_{2}/{l}_{1})}^{2}},\,{\rm{for}}\,{\rm{a}}\,{\rm{prolate}}\,{\rm{spheroid}}\\ {d}_{2}={d}_{3}\end{array}$$

2d $$\begin{array}{c}{d}_{1}=1-2{d}_{2}=\frac{1}{{\varepsilon }^{2}}\{1-\frac{\sqrt{1-{\varepsilon }^{2}}}{\varepsilon }{\sin }^{-1}\varepsilon \},\varepsilon =\sqrt{1-{({l}_{1}/{l}_{2})}^{2}},\,{\rm{for}}\,{\rm{an}}\,{\rm{oblate}}\,{\rm{spheroid}}\\ {d}_{2}={d}_{3}\end{array}$$

2e $${d}_{1}={l}_{2}/({l}_{1}+{l}_{2}),{d}_{2}={l}_{1}/({l}_{1}+{l}_{2}),{d}_{3}=0,{\rm{for}}\,{\rm{elliptical}}\,{\rm{cylinder}}$$

According to the concept of neutral inclusion, three equations are obtained by setting ${\kappa }_{1}^{\ast }={\kappa }_{2}^{\ast }={\kappa }_{3}^{\ast }=\kappa$, based on which the material parameters and the geometrical parameters of the coated ellipsoid can be defined precisely. The coated ellipsoid is transparent in the matrix material when the thermal conductivity *κ* = *κ*_0_; otherwise, it is disguised as an ellipsoid camouflage with a thermal conductivity $\kappa$. Without loss of generality, assume that the material parameters *κ*,*κ*_I_, *κ*_II_ and the geometrical parameters *l*_*cj*_ (*j* = 1,2,3) are known; thus, the geometrical parameters *l*_*ej*_ (*j* = 1,2,3) of the coating can be deduced by the three equations. The general conditions of a sphere, a cylinder, a spheroid and an elliptical cylinder camouflage device are derived as stated in Eq. (3) based on Eq. (1) and ${\kappa }_{1}^{\ast }={\kappa }_{2}^{\ast }={\kappa }_{3}^{\ast }=\kappa ,$

3a $${d}_{c{\rm{1}}}-{f}_{{\rm{I}}}{d}_{e{\rm{1}}}={d}_{c{\rm{2}}}-{f}_{{\rm{I}}}{d}_{e{\rm{2}}}$$

3b $${\kappa }_{{\rm{I}}{\rm{I}}}+\frac{{f}_{{\rm{I}}}({\kappa }_{{\rm{I}}}-{\kappa }_{{\rm{I}}{\rm{I}}}){\kappa }_{{\rm{I}}{\rm{I}}}}{{\kappa }_{{\rm{I}}{\rm{I}}}+({d}_{c1}-{f}_{{\rm{I}}}{d}_{e1})({\kappa }_{{\rm{I}}}-{\kappa }_{{\rm{I}}{\rm{I}}})}=\kappa $$

Therefore, the geometrical parameters *l*_*ej*_ (*j* = 1,2,3) of the coating can be calculated by inserting Eq. (2) into Eq. (3) in a MATLAB program. We have to emphasize that there is a solution of *l*_*ej*_ only when the thermal conductivity *κ* *∈* [*κ*_I_ *, κ*_II_] or*κ* *∈* [*κ*_II_ *, κ*_I_]. According to the composites theory, the thermal camouflage device can be effective in the transient regime when the product of density and heat capacity of the cloak satisfies the relationship *ρC* = *(ρC*)_II_(1-*f*_I_) + (*ρC*)_I_ *f*_I_. Because it is difficult even impossible to find the natural materials which satisfy the thermal conductivity condition and the *ρC* condition simultaneously, here we only consider the thermal conductivity condition and the influence of *ρC* on the transient effectiveness will be analysed later.

**Figure 2:**
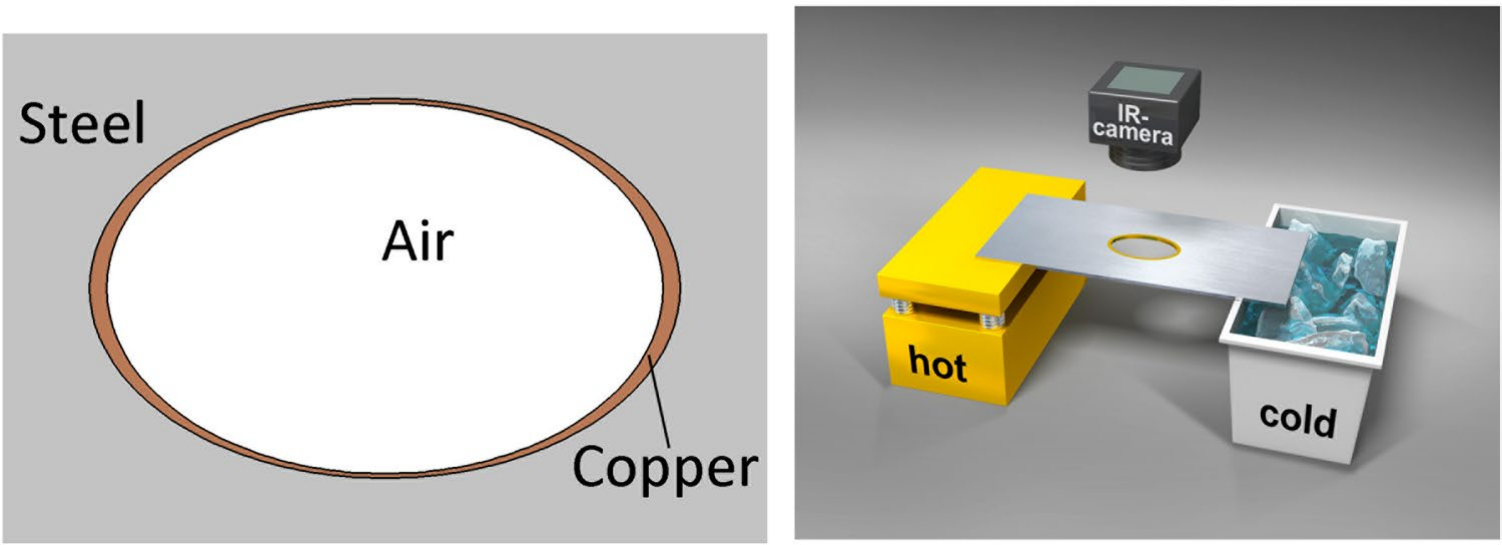
Schematic Illustration of the Elliptical Thermal Cloak with Naturally Available Materials (**a**), and the setup of the experiment (**b**).

Because the invisible device is a special case of camouflage devices, transient experiments of an invisible device when *κ* = *κ*_0_ are conducted in this Letter. To be thermally invisible, an elliptical air hole with ${l}_{c1}=3\,{\rm{cm}}$ and ${l}_{c2}=2\,{\rm{cm}}$ in a steel plate is cloaked by copper. Hence, the background, the elliptical core and the exterior cloaking are steel, air and copper, respectively; that is, $\kappa ={\kappa }_{{\rm{0}}}=\mathrm{16}{\rm{.2}}\,{\rm{W}}/{\rm{mK}}$, *κ*_I_ = 0, and ${\kappa }_{{\rm{II}}}=385{\rm{W}}/{\rm{mK}}$. According to Eq. (3), the geometrical parameters of the copper cloaking are computed to be ${l}_{e1}=3.187\,{\rm{cm}}$ and ${l}_{e2}=2.048\,{\rm{cm}}$. The schematic configuration of the elliptical invisible cloak and the setup of the experiment are shown in Fig. 2).

During the experimental period, we place the left and right sides of the fabricated thermal cloak shown in Fig. 2 into an 80 °C hot source achieved with a controllable heater and into a 0 °C cold source maintained by a tank filled with an ice-water mixture, respectively. The real-time temperature profile is monitored using an infrared camera (FLIR E5) at *t* = 2, 4, 6, and 30 min, and the sample tends to reach thermal equilibrium thereafter.

**Figure 3:**
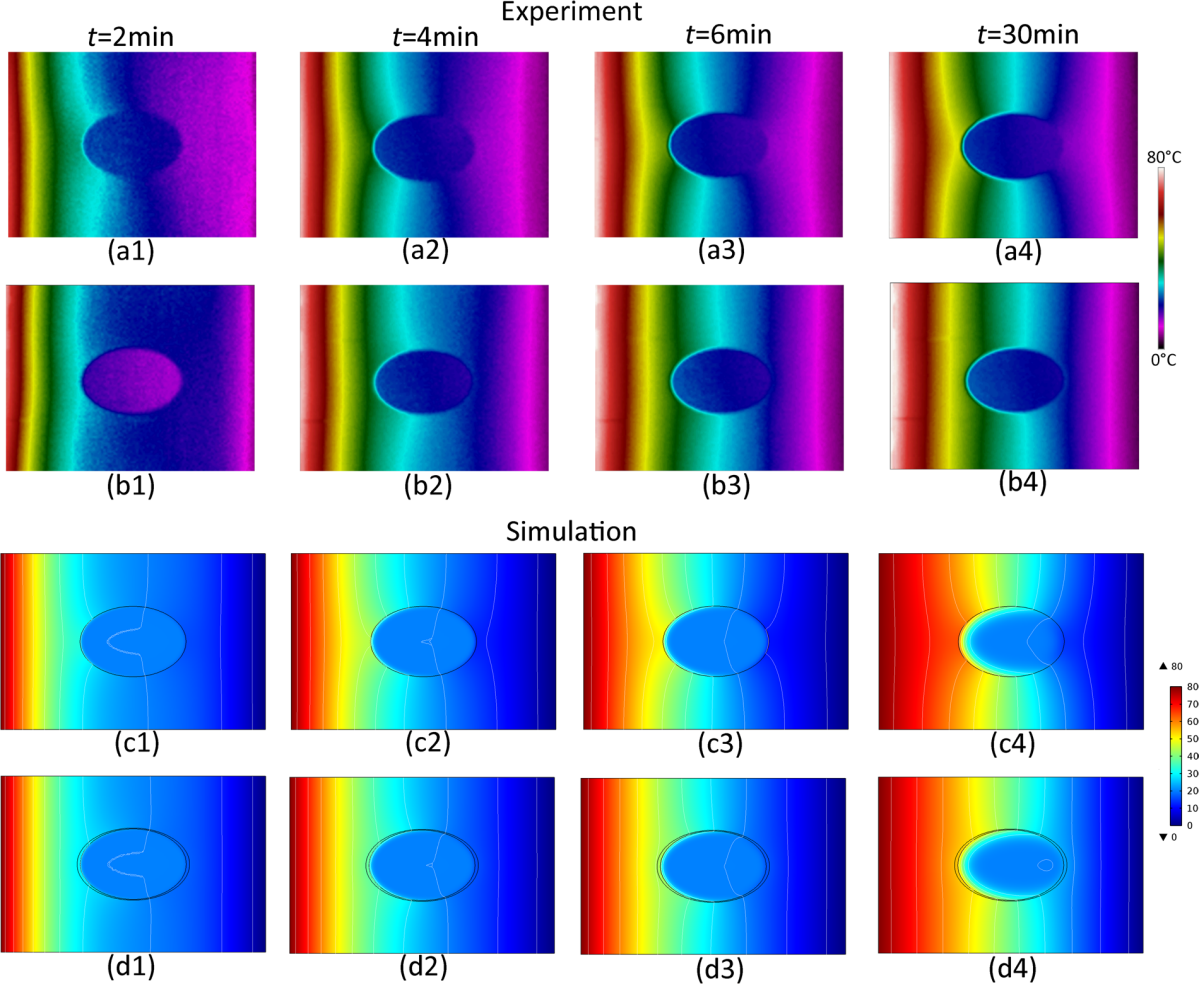
Experimental and Simulated Distributions of the Thermal Field when Heat Flux is Transferred in the Horizontal Direction. The First and the Third Lines Correspond to the Specimen without a Cloak, and the Second and the Fourth Lines Correspond to the Specimen with a Cloak. The Four Columns Represent the Temperature Contours at Times *t* = 2, 4, 6, and 30 Min.

Snapshots of the measured and the simulated transient heat signatures of the fabricated sample are shown in Fig. 3. The first and third lines are the transient distributions of the thermal field of the specimen without a cloak, and the second and fourth lines represent those of the specimen with a cloak. The four columns display the heat signatures at times *t* = 2, 4, 6, and 30 min. Comparing rows (a) and (b) with rows (c) and (d), the experimental results of either the specimen without a cloak or the one with a cloak are in good agreement with the simulated one at each time. Both the experimental and simulated results show that the front of the heat flux remains undistorted by the cloak layers and retains the original direction entirely behind the cloak, thus successfully leaving the thermal flux undisturbed by the protected core. Thus, the existence of the core object will not distort the surrounding heat flux, that is, it is thermally invisible. Such an undisturbed inclusion is very useful and important in the field of integrated circuits with thermal components.

**Figure 4:**
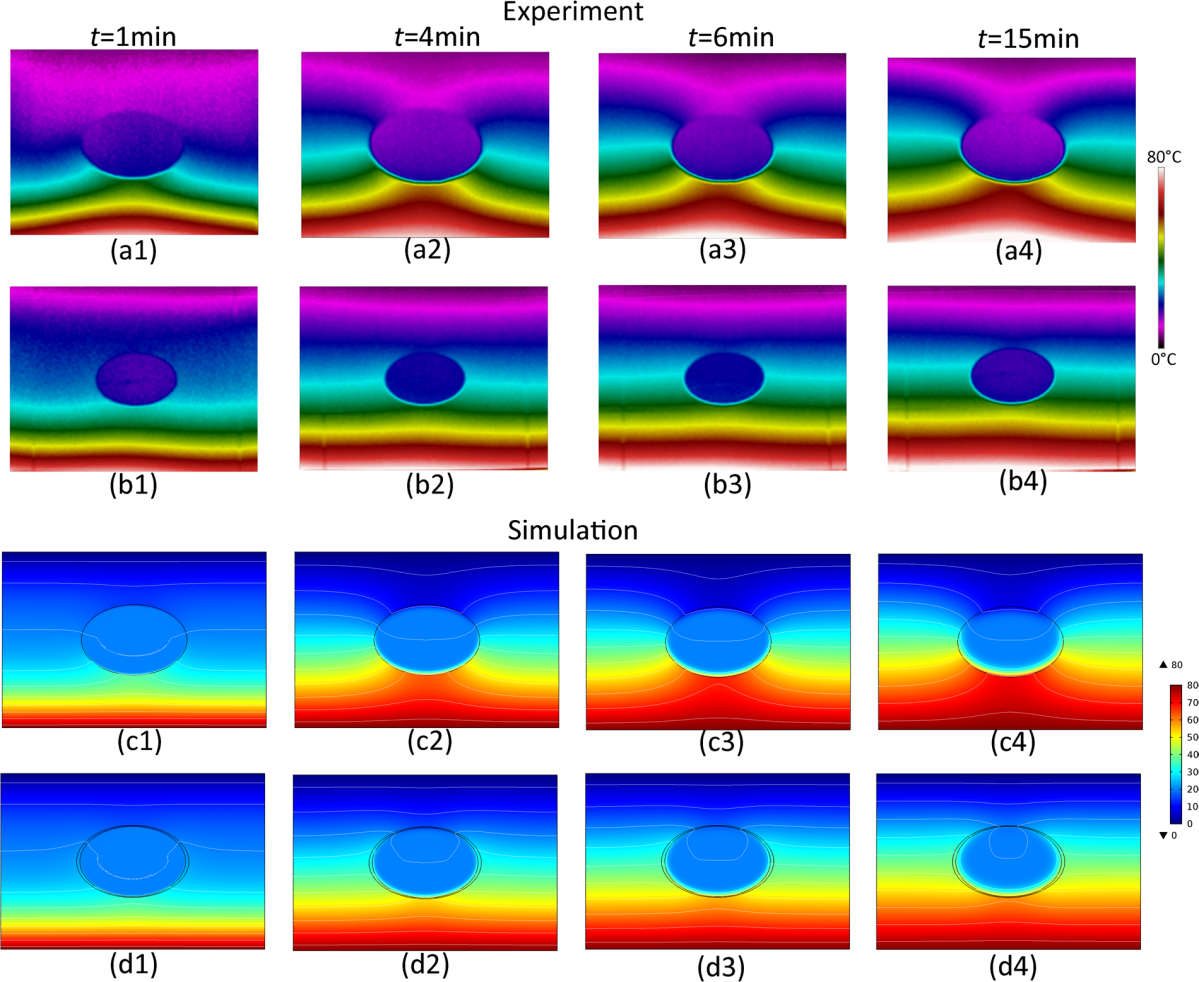
Experimental and Simulated Distributions of the Thermal Field when Heat Flux is Transferred in the Vertical Direction. The First and the Third Lines Correspond to the Specimen without a Cloak, and the Second and the Fourth Lines Correspond to the Specimen with a Cloak. The Four Columns Represent the Temperature Contours at Times *t* = 1, 4, 6, and 15 Min.

To examine the omnidirectional effectiveness of the proposed invisible device, the hot source and the cold source mentioned above are loaded on the bottom and top sides, respectively. Next, the heat flux is transferred in the vertical direction. The real-time temperature profile is monitored at *t* = 1, 4, 6, and 15 min. The corresponding results are displayed in Fig. 4, where each line represents the same specimen as in Fig. 3, and the heat signatures of four columns are at times *t* = 1, 4, 6, and 15 min. As expected, the monitored results not only comply with the simulated results but also indicate that the neutral inclusion is undisturbed in the vertical direction, that is, the elliptical cloak designed here is also effective in the vertical direction. In addition, the simulation of the cloak is conducted when the heat conduction is along different directions (such as pi/6 here). The corresponding calculated results are shown in Supplementary Figure S1. Obviously, both for horizontal incidence (Fig. 2) and vertical incidence (Fig. 4), the contours of the thermal field remain a straight line (almost without any distortion) in the background region.

**Figure 5:**
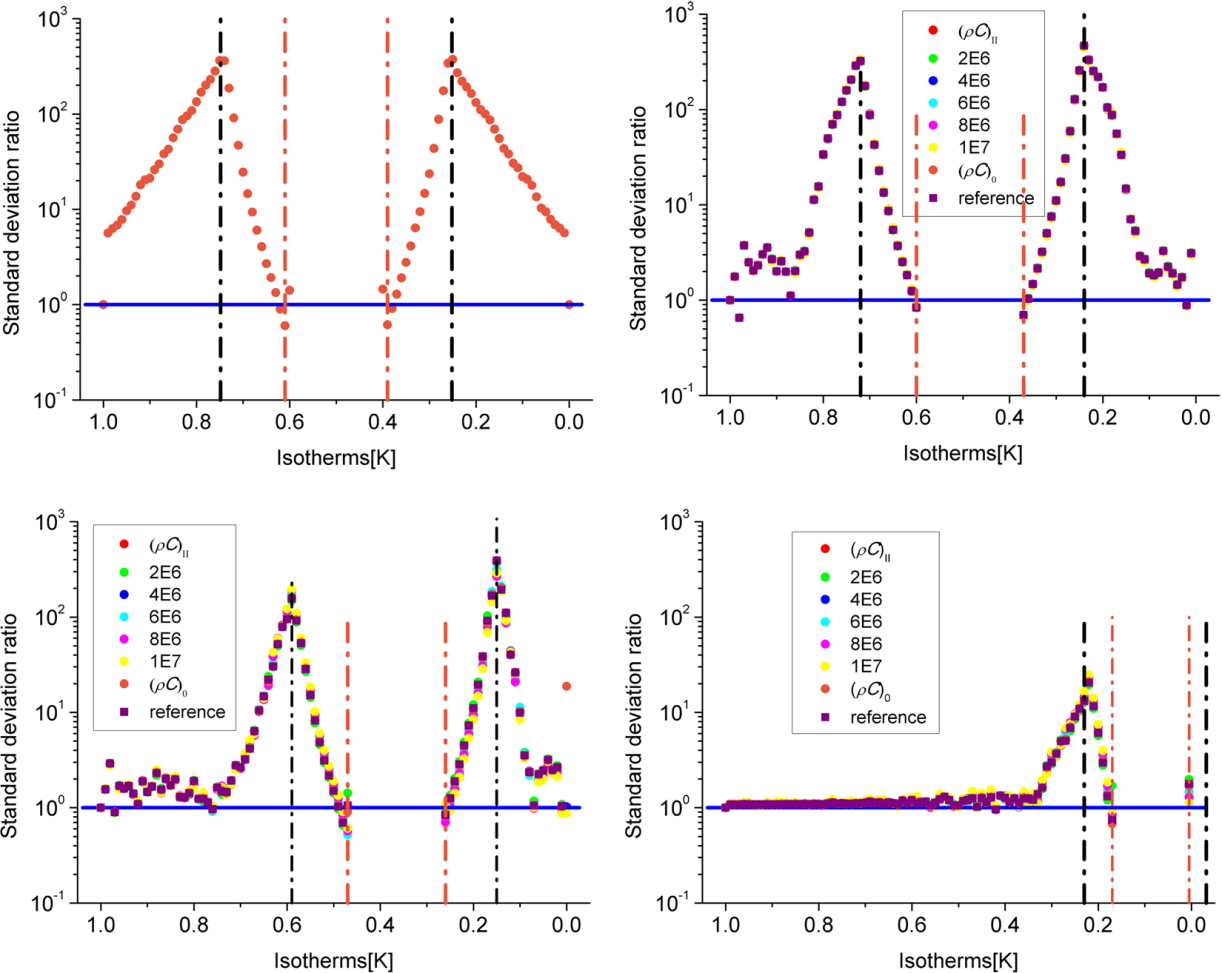
Standard deviation ratio of the invisible thermal cloak at angular frequencies *ω* = 0, 1, 3 and 8 rad.s^−1^. The source is on the left so that heat diffuses from left to right. The vertical lines are related to the isotherms on the boundary of the object without a cloak (black) and with a cloak (orange). The vertical axis gives the ratio of the deviation on a logarithmic scale, while the horizontal axis is for the temperature of the isotherms. Note that this is no longer the case at increasing frequencies, which explains the shift of the curves with frequency *ω*.

Although we did not discuss the product of density and heat capacity, the invisible thermal cloak designed above works well in the transient regime. Next we will give the explanation. The products of densities and heat capacities for the background, the elliptical core and the exterior cloaking are (*ρC*)_0_ = 3.98 × 10^6^ J/Km^3^, (*ρC*)_I_ ≈ 1.23 J/Km^3^, and (*ρC*)_II_ = 3.42 × 10^6^ J/Km^3^, respectively. Actually, *ρC* of the coat should be 4.93 × 10^6^ J/Km^3^ based on the composites theory, which named reference here. To evaluate quantitatively the influence of the product *ρC* on the transient effectiveness of the invisible thermal cloak, the standard deviation ratio of the isotherms is calculated by using the method proposed in ref.^28^. Several corresponding standard deviation ratios can be achieved by changing the value of (*ρC*)_II_, which are displayed in Fig. 5. The standard deviation ratio of isotherms is defined by *std*(bare object)/*std*(studied cloak), where *std*(bare object) denotes the standard deviation of the isotherms when heat diffuses through the bare object to be cloaked and *std*(studied cloak) is the standard deviation of the isotherms when the object is coated by the invisible cloak. An efficient cloak means that *std*(studied cloak) is close to 0. Therefore the standard deviation ratio increases with increasing effectiveness. In Fig. 5, it is easy to find that all curves are above the horizontal blue line which is a hallmark of neutral effectiveness and overlap with each other, that is to say, the spectral effectiveness of the invisible thermal cloak is close to perfect and almost has nothing with the product (*ρC*)_II_. It may be because the core is air which can be looked as a vacuum hole and the coat layer is very thin, so the influence of the product (*ρC*)_II_ is weak and unobvious.

For a real camouflage device, the surrounding material is filled by an alloy (grade 6061), and the disguised object is Cu, the illusion object is Pb, and the coating is steel (grade 304). Experiments in both the horizontal and vertical directions are conducted. The monitored and corresponding simulated results are shown in Figures S2–S5. The omnidirectional effectiveness of the proposed camouflage device can also be examined by calculating the oblique incidence cases shown in Figure S6. It is concluded that the camouflage device functions equally well for arbitrary incident heat fields via the equivalence observed in the identical patterns of the second and the third columns in Figure S2 for horizontal incidence, in Figure S4 for vertical incidence, and in Figure S6 for oblique incidence. The thermal scattering pattern of camouflage agrees very well with corresponding expected objects regardless of the incident direction. However, although the thermal camouflage device is nearly perfect according to the qualitative research, it is an approximate transient device because of the deviation of the product (*ρC*)_II_ (3.9809 × 10^6^ J/Km^3^) from the referenced one (1.9 × 10^5^ J/Km^3^) calculated by the composites theory. The quantitative analysis of the standard deviation ratio of the isotherms is conducted to discuss the influence of the product (*ρC*)_II_ on the transient performance of the thermal camouflage, which is figured out in Figure S7. The standard deviation ratio is defined by *std*(illusion object)/*std*(studied camouflage device), where *std*(illusion object) denotes the standard deviation of the isotherms when heat diffuses through the illusion object and *std*(studied camouflage device) is the standard deviation of the isotherms when heat diffuses through the studied camouflage device. An efficient camouflage device means that the standard deviation ratio is close to 1. The transient performance near the boundary of the illusion object is weakened by the deviation of (*ρC*)_II_ from the reference, and this disturbance will be enhanced via the increase of the angular frequency. That means the transient performance is not perfect at the beginning of the heat transfer and tends to be perfect with the increase of time when (*ρC*)_II_ is different from the reference.

## Conclusion

In summary, we have demonstrated a thermal camouflage device that enables changes of the thermal signatures for one object into another in a specific physical field. Our design scheme, which is based on the idea of neutral inclusion, does not rely on transformation optics, and thus avoids the problems of previous proposals, such as extreme parameters (inhomogeneous and anisotropic material properties) and complicated fabrication processes. In addition, the one heat-insulated layer here is more realizable than the similar device derived from a bilayer cloak that was proposed in ref.^19^. The metamorphosis of the thermal signature has been verified for invisible cloak and camouflage cases under the transient situation, both of which demonstrate good thermal camouflage performance. It should be noted that the camouflage devices presented can be easily realized and experimentally verified. The approximate transient performance can be quantitatively evaluated by the calculating of the standard deviation of the isotherms in advance. In addition, although the neutral conditions given here are only available for the ellipsoid cases, other-shaped thermal camouflage devices can be designed based on the concept of neutral inclusion as well, however, maybe only numerical results rather than analytical equations can be achieved.

## Methods

We introduce the concept of the neutral inclusion into the design of the ellipsoid thermal camouflage device. The geometrical parameters of mentioned models and the standard deviation ratios in this paper are calculated numerically by MATLAB (R2010a). Numerical simulations are performed with heat transfer module of COMSOL Multiphysics (the commercial software package based on finite-element method) where simulated models and boundary conditions are as the same as used in the experiments. Top surfaces of experimental models are coated by black electrical tape which is nearly “black” for the wavelength seen by the thermal heat camera.

### Data availability statement

The authors declare that there is no restriction on the availability of materials or information and all materials, data and associated protocols are promptly available to readers without undue qualifications in material transfer agreements.

## Electronic supplementary material

Supplementary Information
