# Supplementary material for: Transient experimental demonstration of an elliptical thermal camouflage device
Source: Sci Rep. 2017 Nov 30;7:16671. doi: 10.1038/s41598-017-17016-7 (PMC5709401; doi:10.1038/s41598-017-17016-7)
Supplement: Supplementary file 1 — Supplementary Information [file 41598_2017_17016_MOESM1_ESM.doc]

Supporting Information

Transient Experimental Demonstration of an Elliptical Thermal Camouflage Device

Xiao He, Tianzhi Yang, Xingwei Zhang, Linzhi Wu and Xiao Qiao He^*^


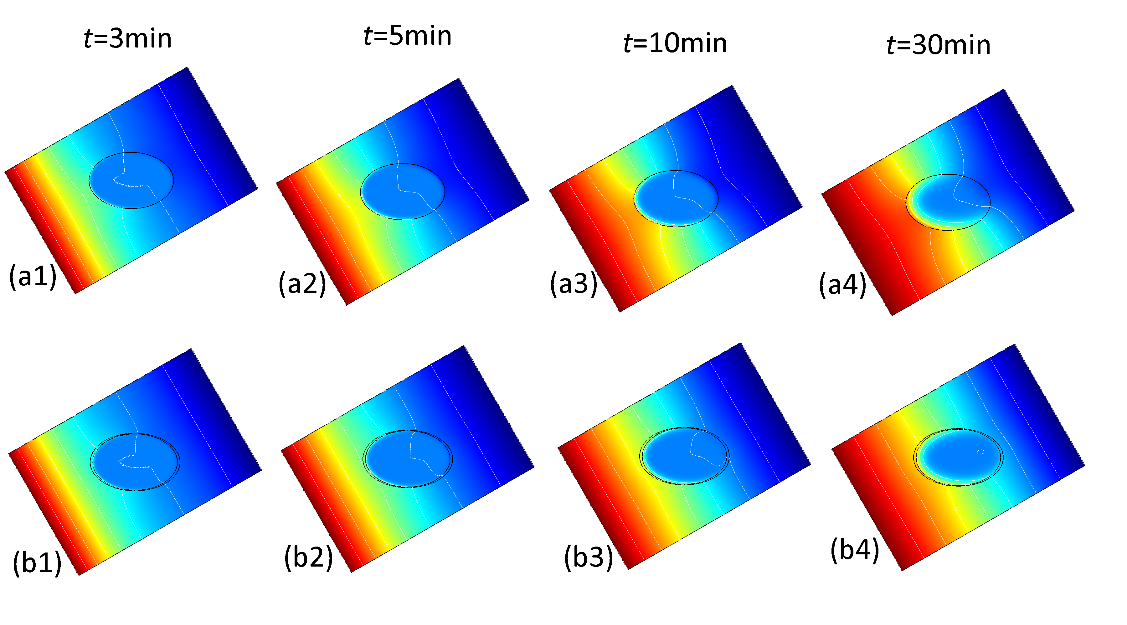


**Figure S1.** Simulated Contours of the Thermal Field at Times t=3, 5, 10, and 30 min. (a1)-(a4) are the simulated results of a steel plate including an air hole, and (b1)-(b4) are the simulated results of the corresponding model cloaked by an elliptical Cu shell.


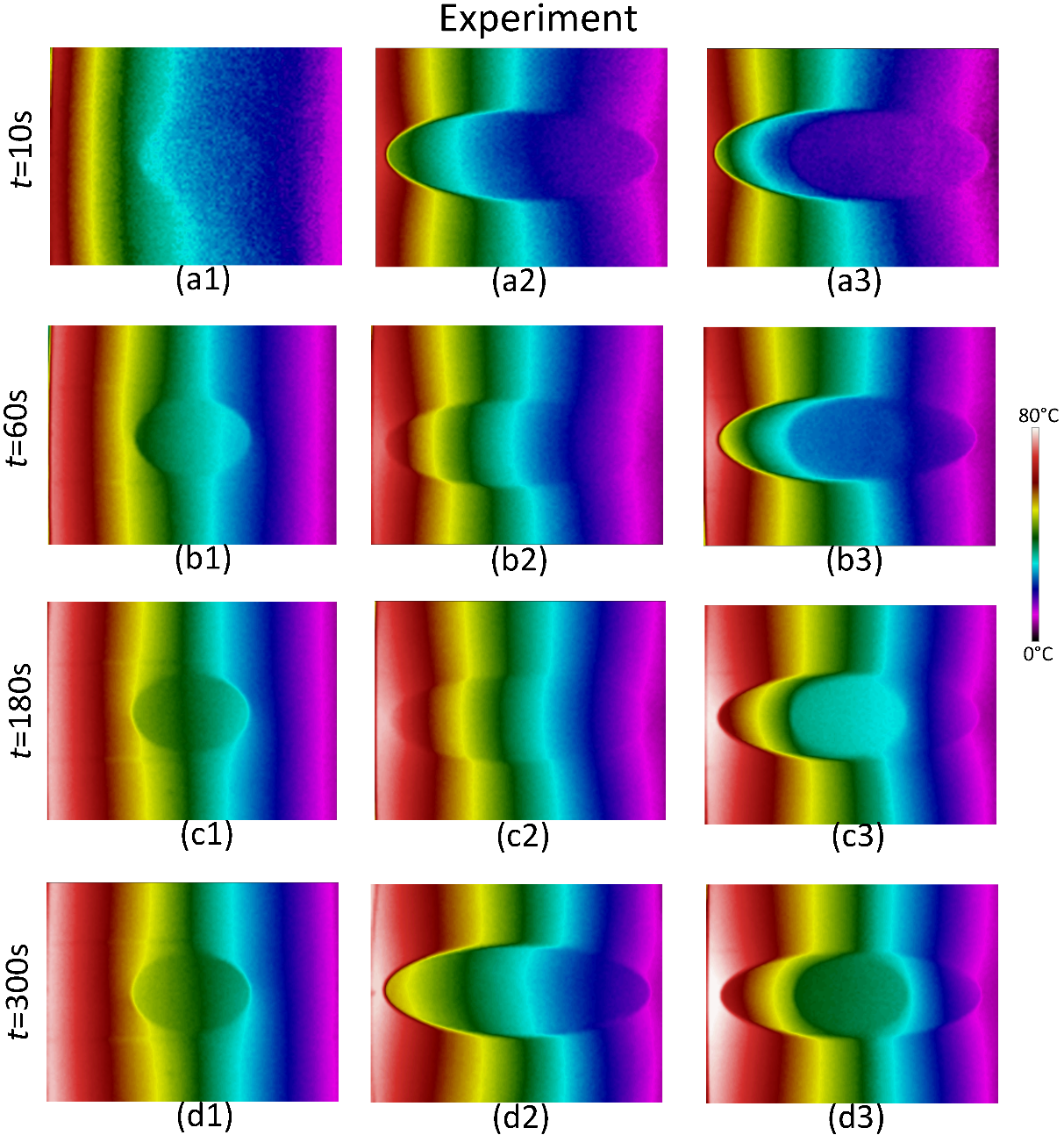


**Figure S2**. Transient Experimentally Monitored Contours of the Temperature Distribution at Times t=10, 60, 180, and 300 s when the Thermal Flux Flows in the Horizontal Direction. The Columns are the Corresponding Results of the Al Plate with an Elliptical Inclusion Cu, with an Elliptical Inclusion Pb, and with a Coated Inclusion Cloaked by an Elliptical Steel Shell.


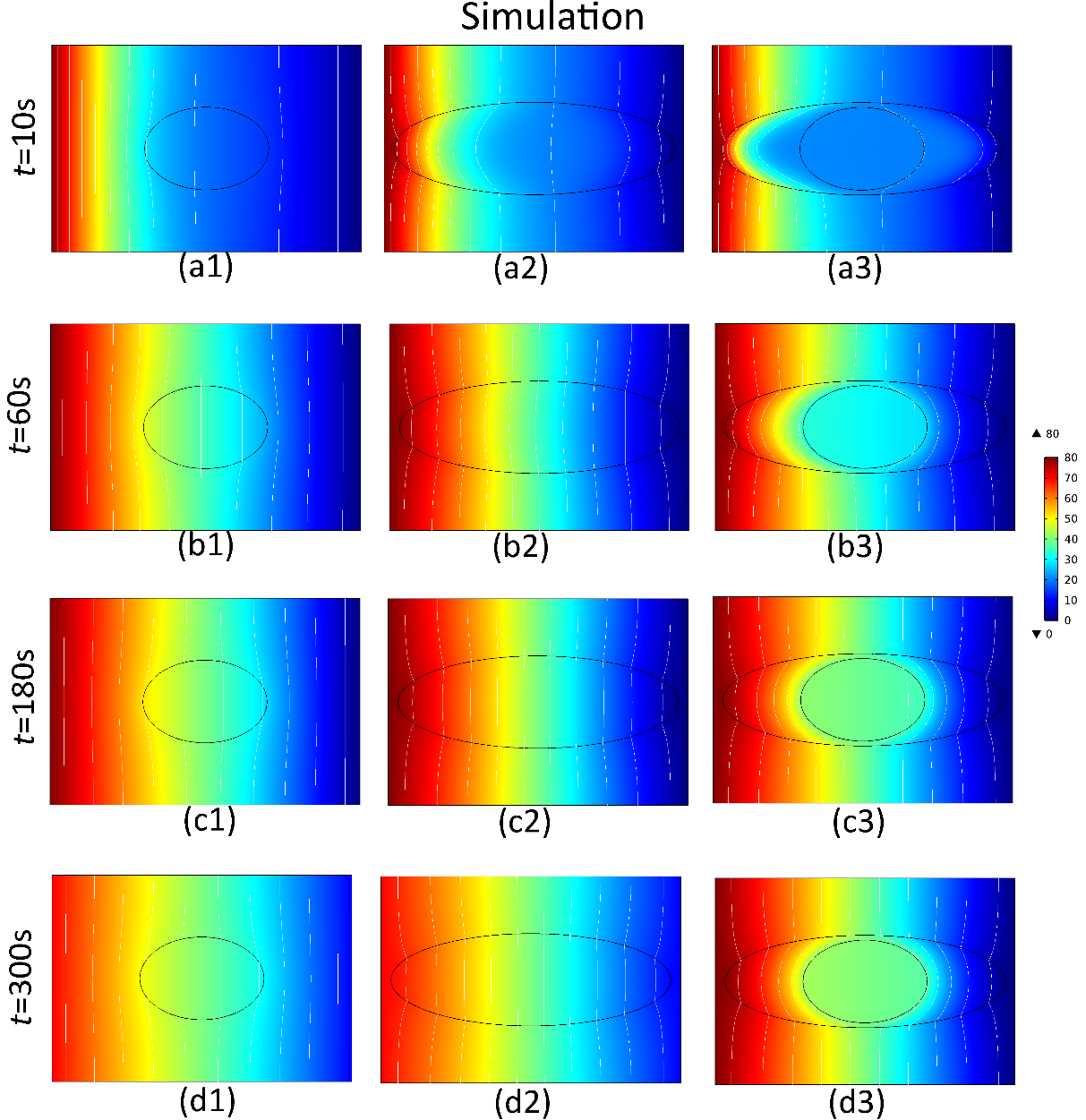


**Figure S3** Transient Simulated Contour of the Temperature Distribution at Times t=10, 60, 180, and 300 s when the Thermal Flux Flows in the Horizontal Direction. The Columns are the Corresponding Results of the Al Plate with an Elliptical Inclusion Cu, with an Elliptical Inclusion Pb, and with a Coated Inclusion Cloaked by an Elliptical Steel Shell. The White Lines are the Isothermal Lines.


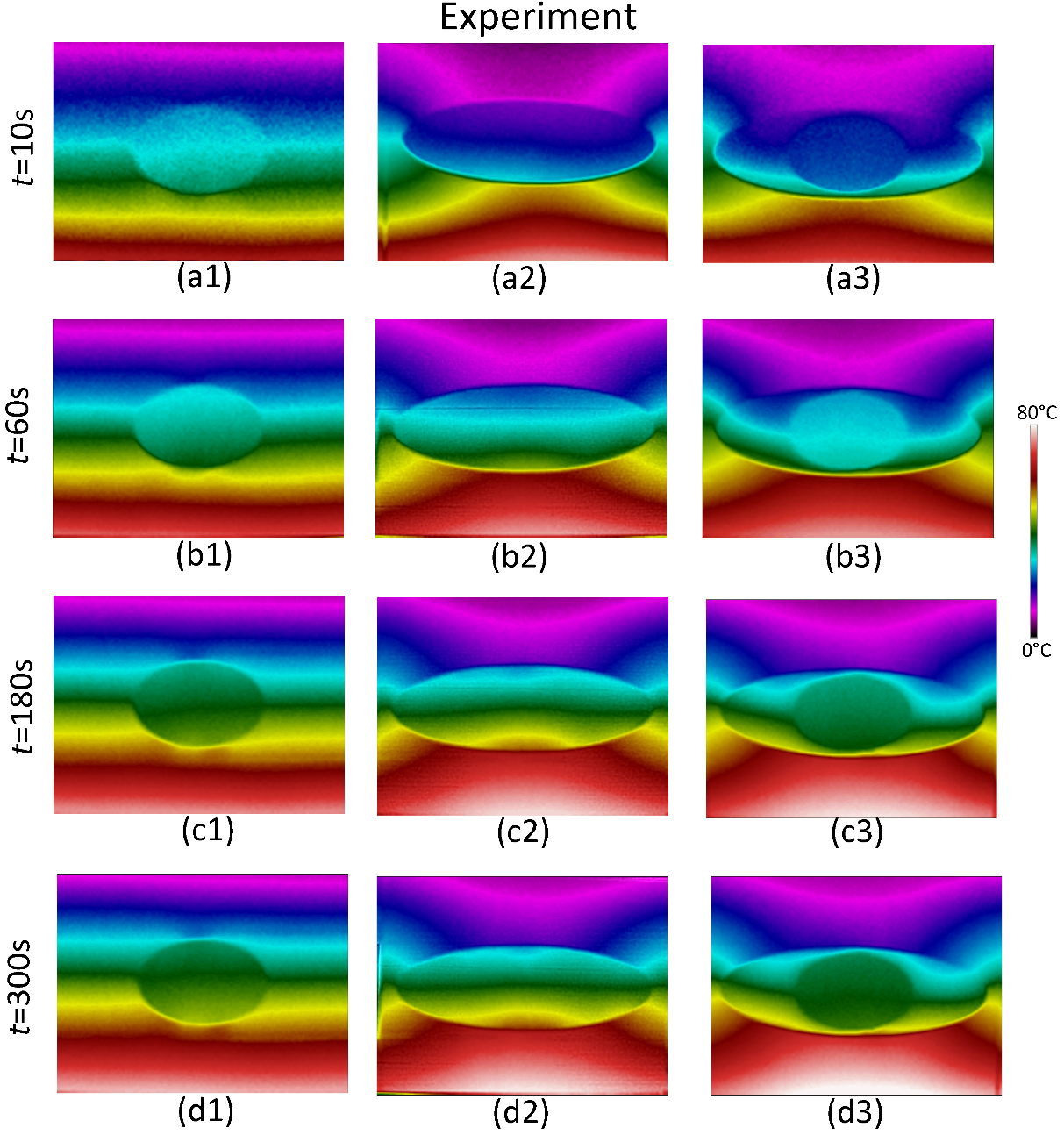


**Figure S4**. Transient Experimental Monitored Contour of the Temperature Distribution at Times t=10, 60, 180, and 300 s when the Thermal Flux Flows in the Vertical Direction. The Columns are the Corresponding Results of the Al Plate with an Elliptical Inclusion Cu, with an Elliptical Inclusion Pb, and with a Coated Inclusion Cloaked by an Elliptical Steel Shell.


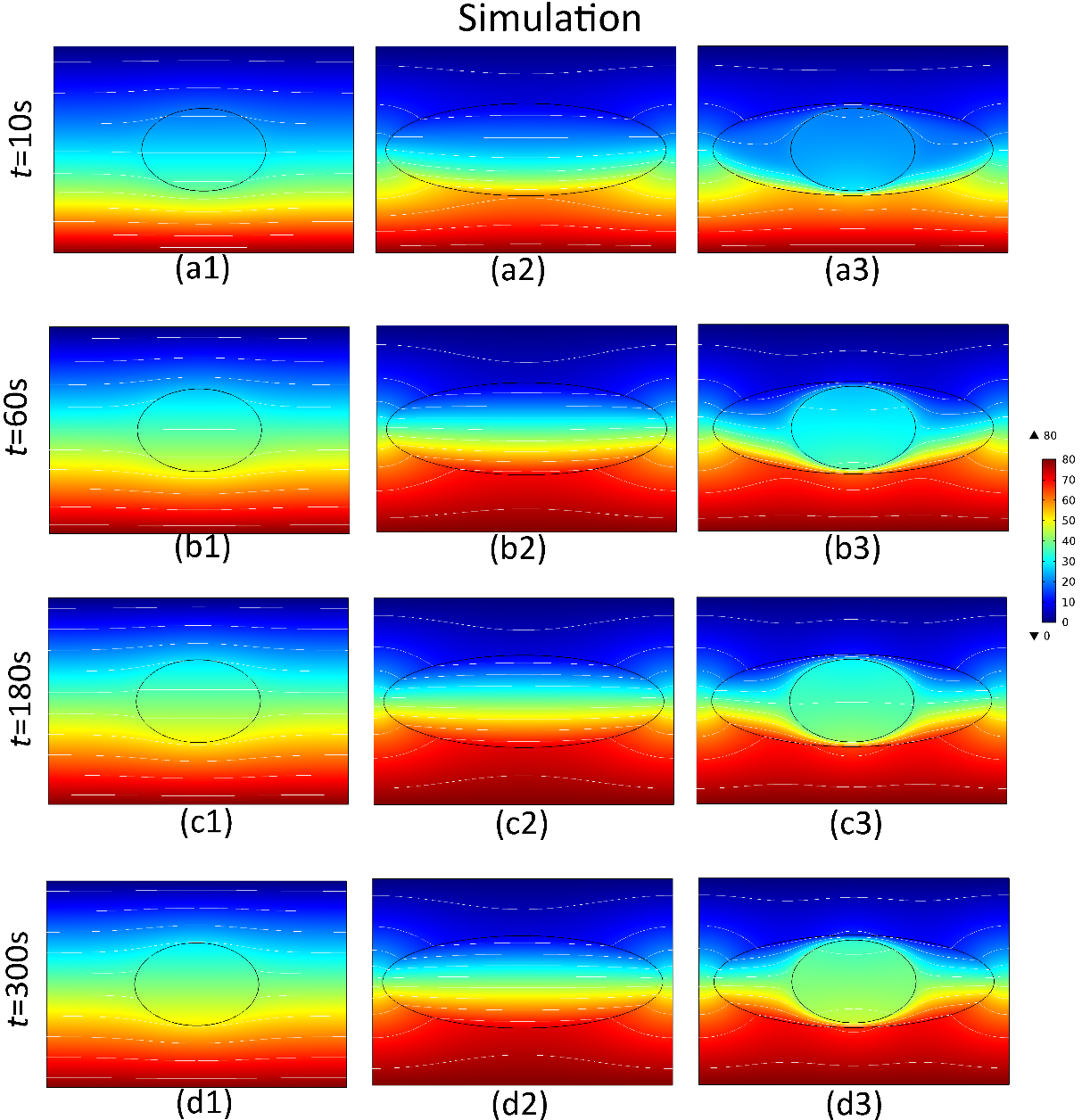


**Figure S5**. Transient Simulated Contour of the Temperature Distribution at Times t=10, 60, 180, and 300 s when the Thermal Flux Flows in the Vertical Direction. The Columns are the Corresponding Results of the Al Plate with an Elliptical Inclusion Cu, with an Elliptical Inclusion Pb, and with a Coated Inclusion Cloaked by an Elliptical Steel Shell. The White Lines are the Isothermal Lines.


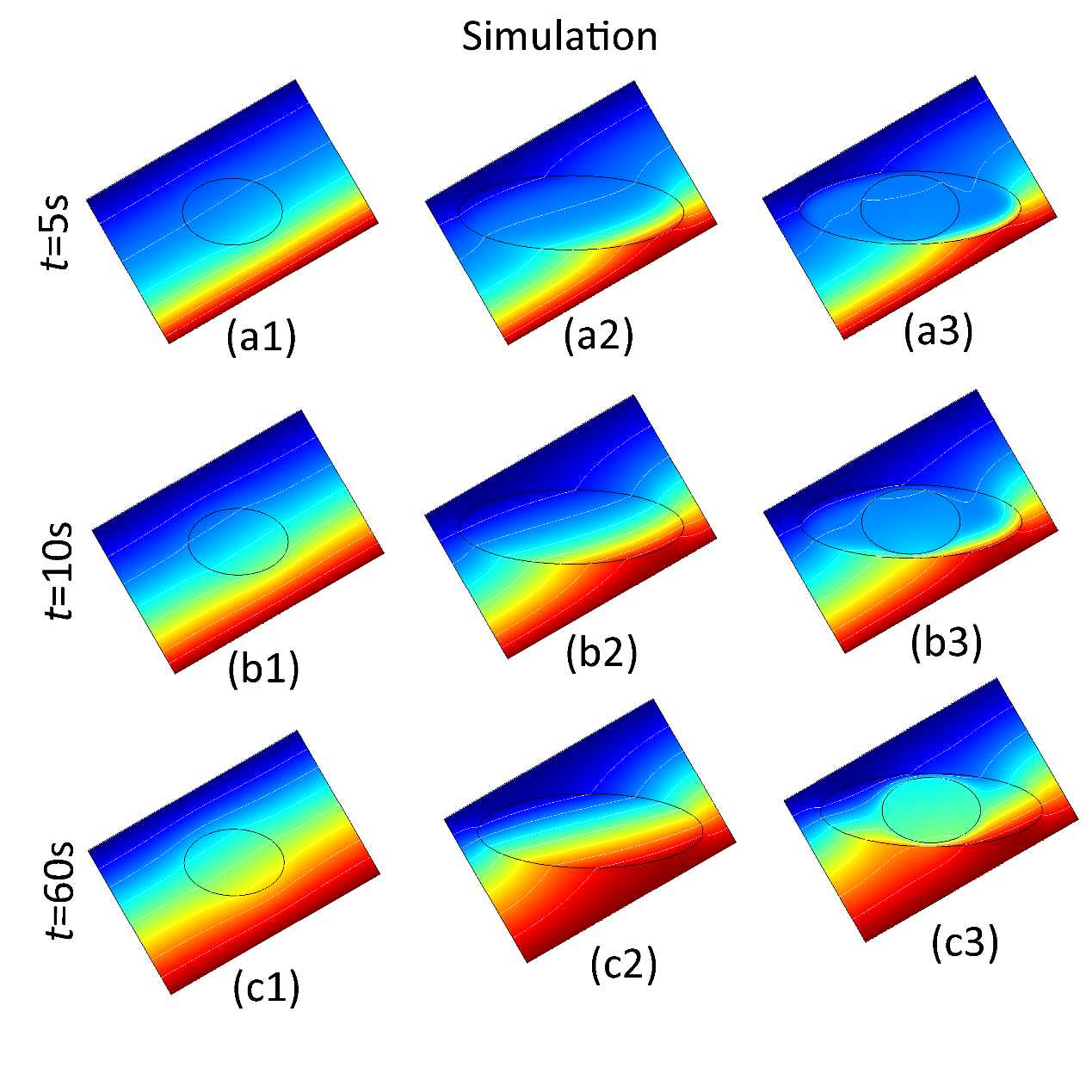


**Figure S6**. Transient Simulated Contour of the Temperature Distribution at times t=5, 10, and 60 s when the Thermal Flux Flows in the 5pi/6 Direction. The Columns are the Corresponding Results of the Al Plate with an Elliptical Inclusion Cu, with An Elliptical Inclusion Pb, and with a Coated Inclusion Cloaked by an Elliptical Steel Shell. The White Lines are the Isothermal Lines.
